# Challenges and barriers to HIV care engagement and care cascade: viewpoint

**DOI:** 10.3389/frph.2023.1201087

**Published:** 2023-07-20

**Authors:** Khayreddine Bouabida, Breitner Gomes Chaves, Enoch Anane

**Affiliations:** ^1^ Research Center of the Hospital Center of the University of Montreal (CRCHUM), Montreal, MTL, Canada; ^2^ École de Santé Publique, Université de Montréal, Montreal, QC, Canada; ^3^ Department of Biomedical Research, St. George’s University School of Medicine, Great River, NY, United States; ^4^ Department of Public Health Research, Vitalité Health Network, Moncton, NB, Canada

**Keywords:** human immunodeficiency virus (HIV), patients, HIV care cascade, challenges, patient engagement (PE)

## Abstract

Patients with human immunodeficiency virus (HIV) are subject to long-term management and a complex care process. Patients with HIV are clinically, socially, and emotionally vulnerable, face many challenges, and are often stigmatized. Healthcare providers should engage them with diligence in the HIV care cascade process. In this paper, we discuss from our viewpoint certain social and public health barriers and challenges that should be considered by healthcare providers to better engage patients in the HIV care cascade process and maximize its outcomes.

## Introduction

HIV (Human Immunodeficiency Virus) infection is a viral infection caused by the HIV virus (1–8). HIV primarily targets and attacks the immune system, specifically the CD4 cells (also known as T-helper cells) that play a crucial role in coordinating the immune response against infections. When a person becomes infected with HIV, the virus invades body tissues and replicates inside CD4 cells (1–8). As the virus multiplies, it progressively weakens the immune system, making the person more susceptible to various infections and diseases (1–8). Over time, HIV infection can progress to a more advanced stage known as AIDS (acquired immunodeficiency syndrome) (1–8). HIV is transmitted through certain body fluids, including blood, semen, vaginal fluids, rectal fluids, and breast milk (8–14). HIV infection progresses through different stages (1, 2, 8–14). The first is the acute HIV infection stage, and this is the initial stage immediately after infection, when the virus replicates rapidly (1, 8). The patient may experience flu-like symptoms such as fever, fatigue, sore throat, swollen lymph nodes, and rash. Chronic HIV infection emerges after the acute stage, HIV enters a chronic or latent phase where the virus continues to replicate at a slower pace (1, 8). Patients may not have any symptoms during this phase, but the virus is still active and gradually depleting CD4 cells (1, 2, 8–14). In the late stage, which is a continuation of the chronic stage without treatment, HIV infection progresses to AIDS, which is characterized by severe immune system damage (1, 8). Patients with AIDS are at high risk of developing opportunistic and certain cancers (1, 2, 8–14). Diagnosis of HIV infection is typically done through blood tests that detect the presence of HIV antibodies or the virus itself (1, 2, 8–14). Early diagnosis is important as it allows for timely initiation of antiretroviral therapy (ART), which can effectively control the virus, slow disease progression, and reduce the risk of transmission (1, 2, 5–8). HIV infection is a lifelong chronic condition requiring long-term management and a complex care process. but with appropriate medical care, including ART, patients with HIV may lead to healthy lives, maintain an average life expectancy, and significantly reduce the risk of transmitting the virus to others (1, 8, 15–20). The Long-term management and complex care for patients with HIV should focus on several key factors (1, 2, 8–14). Antiretroviral Therapy (ART) and continuous adherence to ART is essential for the long-term management of HIV (21–26). ART as a standard treatment for HIV infection, is used to suppress the replication of the HIV in the body. The primary goal of ART is to achieve and maintain viral suppression, where the level of HIV in the blood becomes undetectable or very low. By reducing the amount of HIV in the body, ART helps to slow down the progression of HIV infection, preserve immune function, and prevent the development of AIDS. ART significantly improves the health outcomes and quality of life for people living with HIV (1, 2, 8–14). Viral suppression achieved through ART allows the immune system to recover and reduces the risk of opportunistic infections and AIDS-defining illnesses. ART helps to maintain and increase CD4+ T cell counts, which are crucial for a healthy immune system. Furthermore, ART can also reduce the risk of HIV transmission to sexual partners, as having an undetectable viral load significantly lowers the risk of transmission (1, 2, 8–10). Another important factor is the regular medical monitoring of patients with HIV which requires frequent monitoring of their CD4+ T cell count, viral load, and overall health. This includes routine laboratory tests, physical examinations, and screenings for co-infections, such as hepatitis and sexually transmitted infections. HIV management should include preventive measures such as vaccinations (e.g., influenza, pneumococcal vaccines), screening for and treatment of co-infections, and counseling on safe sex practices to prevent transmission of HIV and other sexually transmitted infections (1, 8–14). Moreover, comprehensive support is crucial for long-term care for patients with HIV and should involve a multidisciplinary approach, including access to healthcare professionals, such as infectious disease specialists, HIV specialists, psychologists, social workers, and support groups. Mental health support and addressing social determinants of health (e.g., stigma, discrimination, housing, employment) are all crucial for overall well-being. Finally, patient engagement is essential to providing education and support to help patients understand their condition, manage medication regimens, and fully adhere to the HIV care cascade (1–3, 8). The HIV care cascade, also known as the care continuum, focuses on patient engagement in the HIV care process (1–3). The HIV care cascades through patient engagement aims to enhance ART's impact and improve patient health and well-being (1, 2, 5–8). This approach includes diagnosis, initial patient integration to care (including psychological support), reinforcing adherence, and antiretroviral therapy (ART) maintenance (21–26). In this context, this paper discusses from our perspective that is based on our experience in public health but also within the clinical settings and research in the domain of HIV and patient engagement supported but certain literature references, some of the most important aspects, barriers and challenges that may hinder the engagement of patients and the HIV care cascade. We also formulate, when possible, some recommendations that health systems and healthcare providers may need to consider when implementing patient engagement strategies to improve outcomes.

### Stigma and vulnerability

One of the most critical and common barriers to engaging patients with HIV is stigma (6–10, 26). Stigma can arise from the external environment, known as social stigma, or from within patients living with HIV themselves, referred to as self-stigma or internalized stigma (6–10, 26). Social stigma refers to society's negative attitudes, beliefs, and discriminatory behaviours towards people living with HIV. It can manifest in various ways, including fear, prejudice, avoidance, gossip, rejection, and discrimination towards patients with HIV. Stigmatizing attitudes and actions can be driven by misinformation, misconceptions, fear of transmission, moral judgments, and cultural or religious beliefs. Social stigma can result in isolation, social exclusion, loss of social support, diminished self-esteem, and restricted opportunities for employment, education, and personal relationships. It can lead to reluctance in seeking HIV testing, disclosing HIV status, or accessing healthcare services, hindering timely diagnosis and engagement in care (6–10, 26). Self-stigma or internalized stigma which refers to the acceptance and internalization of societal negative attitudes and beliefs about HIV by patients. This type of stigma occurs when patients with HIV adopt and believe the stigmatizing messages they encounter in society, resulting in negative self-perception and lowered self-worth. Patients with self-stigma may feel shame, guilt, self-blame, or a sense of worthlessness, leading to self-isolation, diminished self-care, and avoidance of social interactions or disclosure. This can eventually affect treatment adherence and engagement in care, as patients may internalize negative beliefs about their ability to manage the disease effectively (21–26). Addressing both social stigma and self-stigma is crucial for patient engagement in the HIV care cascade and to improving the well-being and outcomes of patients and promoting their engagement in care by creating a more inclusive and supportive environment for patients with HIV, enabling them to engage in care and live fulfilling lives (21–26). To address this challenge, healthcare providers should create a safe and supportive environment encouraging patients to participate in their care. Effective patient engagement strategies include education, communication, and support. Education about the disease, its treatment, and its management is essential for patients to understand their condition and make informed decisions about their care. Also, communication between healthcare providers and patients should be open and honest, and patients should be encouraged to ask questions and express their concerns. Programs that provide comprehensive care and support to patients with HIV, including medical care, mental health support, counselling, education, and social services, can improve patient adherence and actively help patients engage in their care process (19–23).

### Mental health issues

Patients with HIV are at increased risk for mental health issues, including depression, anxiety, and substance use disorders (5, 6, 8–12). These conditions can negatively impact patient engagement, making it harder for patients to adhere to treatment and regular medical care. In addition, patients with HIV are vulnerable to addiction and substance abuse. Patients with HIV may experience cognitive impairment, including memory loss and difficulty concentrating, which can be caused by the disease or ART medications’ side effects. It is important for healthcare providers to screen patients with HIV for mental health issues and to provide appropriate support and treatment. The integration of recovery theories, exemplified by Recovery Colleges, can be tailored to deliver targeted programs to patients with HIV. This mental health educational approach holds promise in promoting better mental health outcomes, a vital factor in facilitating therapy compliance and overall patient wellness.

### Access to healthcare

Patients with HIV often face challenges in accessing healthcare for various reasons, such as transportation, lack of insurance, and limited availability of specialized HIV care, but also financial and language barriers (8–12). The access also includes language and cultural barriers. Moreover, the financial cost of healthcare can be a significant barrier for people living with HIV, especially in countries with limited resources. Adapting strategies for more vulnerable groups and respecting minority belief systems should also be a tool for improving accessibility. Finally, drug universality programs should be encouraged, as well as international cooperation for the equitable distribution of new drugs worldwide.

### Health literacy

Health literacy refers to a person's ability to understand and use health information to make informed decisions about their health (8–14). Patients with low literacy may face challenges in understanding medical information, navigating healthcare systems, understanding their treatment options and medication instructions, utilizing healthcare services effectively and making informed decisions about their care (8–16). This can result in delays in seeking medical care and treatment, negatively impacting patient engagement, treatment adherence, and interaction with healthcare providers. To address these health literacy issues, healthcare providers should provide clear, simple, and transparent information to patients with HIV, using plain language and visual aids to enhance understanding. Patients should also be encouraged to ask questions and seek clarification. Besides, integrating patient partner models into healthcare services can lead to closer alignment with patients’ needs. This approach empowers services to provide more comprehensive and patient-centred care (12).

### Collaboration and evaluation

Providing comprehensive care for patients with HIV can be challenging. HIV is a complex disease requiring a collaborative and multidisciplinary care approach (13–16). Physicians and nurses, in the case of HIV management, should collaborate and work closely with social workers, nutritionists, mental health professionals, and other specialists to provide patients with the appropriate care. Healthcare providers should collaborate, gather their efforts and competencies, and continuously measure, evaluate, and improve their HIV care process to address these challenges and provide patients with the support they need (8, 13–16).

### Payment models

Payment models for health care providers can influence the quality of care received by HIV patients (17). Due to the social and clinical complexity of the patients with HIV, fee-for-service payment models should be replaced by value-based health models (VBHM). VBHC programs enable healthcare providers to earn incentives based on their ability to achieve positive patient health outcomes (18). In other words, VBHC emphasizes the importance of value creation in healthcare delivery, supporting optimal patient outcomes, and promoting higher quality care standards.

## Conclusion

The HIV care cascade emphasizes the importance of the engagement of patients at each stage to achieve optimal health outcomes (1–3, 8, 26). Adherence to antiretroviral therapy (ART) is a critical component of the HIV care cascade, as it plays a central role in the combat against the virus, improving immune function, and preventing disease progression (1–3, 26). However, adherence to ART can be challenging due to several factors, such as stigma, literacy, healthcare access, mental health issues, and other socioeconomic factors. Suboptimal adherence to ART can result in virologic failure, drug resistance, and suboptimal health outcomes (19–23, 26). Strengthening the impact of ART involves addressing barriers to care, promoting early diagnosis, improving access to treatment, supporting adherence in HIV care cascade, and providing comprehensive care to enhance the health and well-being of patients. Nevertheless, patient engagement is crucial for successful HIV care cascade outcomes. It is a complex and dynamic approach requiring healthcare providers to fully understand not only the clinical aspect of the patient but also the patient's social, cultural, and environmental background without stigma or prejudgment (19–23, 26). This diligent consideration of the patient's unique circumstances is essential for maintaining adherence and achieving successful care outcomes. In this paper we focused on patient engagement and adherence, although barriers and challenges to adherence to the HIV care cascade do not solely originate from patient engagement. External factors and the participation of other parties, such as policyholders and service providers, can indeed contribute to expansion of these challenges as well (19–23, 26). It is crucial to consider and involve all relevant stakeholders to address these barriers effectively. This includes advocating for supportive policies, promoting health equity, increasing funding for HIV programs, expanding healthcare infrastructure, and providing training and education for healthcare providers and patients (24–26). Collaboration between patients, policymakers, service providers, and community organizations is essential to create an enabling environment that facilitates patient engagement and improves HIV care outcomes. By recognizing the multi-faceted nature of barriers to healthcare access and engaging all stakeholders, we can work towards overcoming these challenges and ensuring that patients with HIV receive the comprehensive care and support they need (24–26).
